# When Your Boss Is Under Pressure: On the Relationships Between Leadership Inconsistency, Leader and Follower Strain

**DOI:** 10.3389/fpsyg.2022.816258

**Published:** 2022-05-27

**Authors:** Laura Klebe, Katharina Klug, Jörg Felfe

**Affiliations:** ^1^Department of Work, Organizational and Economic Psychology, Helmut Schmidt University, Hamburg, Germany; ^2^Department of Business Studies and Economics, University of Bremen, Bremen, Germany

**Keywords:** leadership, transformational leadership, abusive supervision, stress, inconsistent leadership

## Abstract

It is widely acknowledged that leadership is crucial for follower health. Under stress, positive leader behaviors such as transformational leadership may decrease and the risk of negative behaviors such as abusive leadership may increase. Followers experience these discrepancies in leadership between routine and stressful periods as inconsistent. While positive and negative leadership is generally associated with follower strain, inconsistency may be stressful by itself, because it entails insecurity and unpredictability in the leader-follower relationship. We suggest that the level of perceived inconsistency and volatility in leaders’ behavior across situations is an additional risk factor for follower health. Moreover, we expect perceived inconsistency to be stronger when leaders are strained. This survey study with *N* = 304 employees examines the relationships between leadership inconsistency and leader as well as follower strain from a followers’ perspective. Participants rated their leaders’ transformational and abusive leadership separately for routine and stressful conditions, their leaders’ strain and their own strain. Employees who experienced stronger discrepancies in leadership between routine and stressful conditions, i.e., more inconsistency, experienced more strain. Moreover, from a followers’ perspective, inconsistencies were stronger when leaders were strained. The findings provide evidence that leadership is less stable and consistent than generally assumed and that inconsistency is an additional risk factor. Leader strain may threaten the consistency of leadership and thereby negatively affect follower health.

## Introduction

Previous research shows that not only employees but also their leaders increasingly suffer from psychological strain (Harms et al., 2017). Leaders are typically confronted with stressors such as time pressure, multitasking, interruptions and high workloads, raising their risk of experiencing strain. Leader strain does not only threaten their own health, but also may have a negative impact on their followers. There is substantial evidence that leader strain and affective well-being are associated with follower strain and well-being via crossover (Bakker et al., 2009; Skakon et al., 2010). In this case, leadership can turn out being an additional stressor instead of a resource for followers (Schyns and Schilling, 2013).

Literature suggests that leadership deteriorations may explain negative effects for followers: Findings from cross-sectional studies indicate that leader strain is positively related to negative leadership as leader strain is associated with anger and anxiety (Mawritz et al., 2014), and negatively related to positive leadership (Harms et al., 2017; Kaluza et al., 2019). However, leaders’ level of strain may vary because calm periods may interchange with stressful periods. While leaders have enough resources and capacities to engage in positive leadership and to avoid negative behavior in calm situations, leaders may lack capacities to continuously engage in positive leadership and to avoid negative behavior in stressful periods, so that behavior within leaders may vary depending on the situation (Courtright et al., 2016; Lin et al., 2016). Accordingly, followers may experience pronounced discrepancies in leadership between calm and stressful situations.

In order to better understand the interplay between leader health and leadership, this study investigates inconsistencies in transformational and abusive leadership caused by leader strain. While most of the literature suggests that leadership is a rather stable and constant behavior that is robust across situations and over time (van Dierendonck et al., 2004; Montano et al., 2017), there is a growing interest in changes and even inconsistencies in leadership (McClean et al., 2019). Referring to Mullen et al. (2011), leadership inconsistency describes the variation of leadership behavior within a person, so that the same leader might display different or even contradictory behavior in different situations. Previous diary studies have already shown that leadership even varies on a daily basis (Breevaart et al., 2014; Breevaart and Bakker, 2018). Different periods of high and low levels of strain may cause changes to leadership within leaders as suggested by Harms et al. (2017). Accordingly, followers may experience pronounced discrepancies in transformational and abusive leadership between calm and stressful situations. However, while minor fluctuations in leadership may be tolerable and have less influence on followers, stronger discrepancies may have a crucial influence on follower health, as stronger inconsistencies and unpredictable behavior may cause insecurity, anxiety and feelings of loss of control (de Cremer, 2003; Schyns and Schilling, 2013; Poethke et al., 2021). Therefore, not only the level of transformational or abusive leadership that may be consistently good or bad relates to followers’ health, but the extent of inconsistency may be a stressor in its own right. However, the amount of inconsistency or even contradictory behaviors within leaders has not been addressed in previous literature. Acknowledging the potential risk of experiencing strong inconsistency, the present study investigates leadership inconsistency with regard to employee health.

In past research, potential negative consequences of high discrepancies in leadership for followers have been overlooked so far because most of the evidence regarding leadership and strain is based on averaged evaluations of leadership over a period of time (Harms et al., 2017). Up to now it remains unknown whether the extent of inconsistency in terms of high discrepancies between routine and stressful situations affects follower strain above and beyond absolute leadership levels, and whether followers’ perceptions of leadership inconsistency are related to perceptions of leader strain. It makes a difference for the understanding of leadership and its consequences in how far perceived inconsistencies have an incremental effect on follower health, as unpredictable leadership may lead to increasing insecurity and thus to additional psychological strain. Moreover, it would be plausible that followers experience stronger deteriorations in leadership when they also perceive high strain among their leaders (Harms et al., 2017), as leadership then may be particularly unpredictable.

**FIGURE 1:**
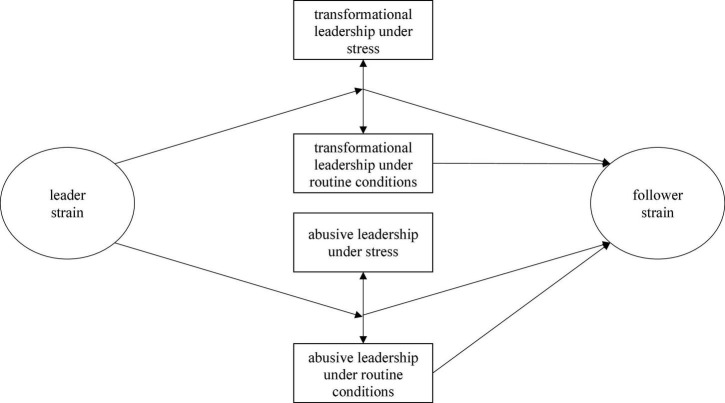
Conceptual model of the study.

As leadership can only affect follower health when it is perceived by followers (Schyns and Schilling, 2013), this study examines followers’ perceptions of leadership inconsistencies in terms of differences in transformational and abusive leadership between stressful and routine situations and their consequences for follower health. We first expect to find systematic differences in both transformational and abusive leadership between routine and stressful situations. Second, and most importantly, we expect that not only the levels of transformational or abusive leadership are crucial for follower health, but that the level of leadership inconsistency (i.e., the extent of discrepancies between routine and stressful periods) has an incremental effect resulting from increased insecurity for followers. This would be in line with literature suggesting negative effects of leadership inconsistency across situations (de Cremer, 2003). Third, based on the literature on leadership deteriorations under stress (Harms et al., 2017), we expect that followers perceive stronger inconsistencies in transformational and abusive leadership when they perceive high strain among their leaders (see our conceptual model in Figure 1). To investigate the relationships between positive and negative leadership, follower and leader health, we focus on follower and leader strain as indicators for their mental health. Moreover, we focus on transformational (Bass, 1998) and abusive leadership (Tepper, 2000) as two contradictory leadership styles in order to contrast effects for outstanding positive and clearly negative leadership behavior. There is ample evidence that these well-established leadership constructs are relevant for follower health.

The findings will contribute to the literature as follows: First, we contribute to the literature on inconsistent leadership by investigating the extent of discrepancies that followers generally perceive among their leaders across situations (de Cremer, 2003; Breevaart and Zacher, 2019). We shift the focus on implications of inconsistency *as such*, in addition to previous studies showing correlations between leadership levels and follower strain in a given situation or time. Second, by investigating leadership inconsistency in terms of differences between routine and stressful situations, the study is the first to investigate the question whether leadership inconsistency has a negative effect for follower health above and beyond absolute leadership levels. Third, the study ties in with research on strain as an important determinant of leadership (Harms et al., 2017; Kaluza et al., 2019) by showing if and to what extent follower perceptions of leader strain relate to perceptions of leadership inconsistency. Comparing behavior within leaders also allows controlling for the influence of stable third variables such as leaders’ personality that may influence both strain reactions and leadership.

## Transformational and Abusive Leadership

Various concepts of leadership and their effects on followers have been discussed in the literature. Previous research suggests that positive leadership styles are positively related to follower health, whereas destructive leadership styles are negatively related to follower health (Skakon et al., 2010; Schyns and Schilling, 2013).

One of the most prominent positive leadership concepts is transformational leadership. Transformational leaders address followers’ values and higher needs to motivate their followers to perform beyond expectation The concept consists of four components: (1) *idealized influence* (i.e., the professional and moral role model function of leaders), (2) *inspirational motivation* (i.e., inspiring employees with attractive and compelling visions), (3) *individualized consideration* (i.e., recognizing and supporting employees’ personal needs), and (4) *intellectual stimulation* (i.e., encouraging employees to think innovatively by constantly questioning previous approaches and trying out new solutions; Bass, 1998). Several studies have shown that transformational leadership has positive effects on employees’ health and well-being (Nielsen et al., 2008), whereas relationships with experienced strain (Felfe, 2006a; Franke and Felfe, 2011), depressiveness (Munir et al., 2012), burnout and emotional exhaustion (Densten, 2005) are negative.

In contrast, negative leadership includes passive as well as active destructive behaviors (Kaluza et al., 2019). One of the most prominent destructive leadership concepts is abusive supervision. Abusive supervision describes the perception of employees of their leader using hostile verbal and non-verbal behaviors excluding physical contact, such as public criticism, rudeness or loud and angry tantrums (Tepper, 2000). Abusive supervision has negative consequences for followers’ health. The literature has shown positive relationships with depression, anxiety symptoms, psychosomatic complaints, emotional exhaustion, burnout, strain and work family conflicts (Wu and Hu, 2009; Schyns and Schilling, 2013), whereas relationships with followers’ mental health and affective well-being are negative (Skakon et al., 2010; Montano et al., 2017).

## Leadership in Calm and Stressful Situations

It is often assumed that leadership is a rather constant behavior. However, there is a growing number of studies indicating that leadership is subject to situational influences (Halverson et al., 2004; Geier, 2016). While leaders can draw on their full psychological capital in calm situations and thus have enough resources to actively engage in constructive leadership, these resources and capacities are limited in stressful periods (Harms et al., 2017). Due to time pressure, leaders may need to shift their focus from their employees to goal orientation and task fulfillment, causing employee-oriented leadership to deteriorate (Hannah et al., 2009; Klebe et al., 2021). Hence, leadership varies across situations, so that leaders likely show less transformational and more abusive leadership in stressful versus routine conditions. In consequence, followers should perceive a clear difference in leadership when their leader is under stress compared to “normal” situations.

First evidence was provided by Geier (2016), who investigated situational effects on within-person changes in leadership. The study showed that transformational leadership is less frequently displayed in extreme contexts, such as firefighting, than under routine conditions. These extreme situations may be also experienced as stressful and thus reduce functional capacity. This supports the assumption that transformational leadership is less pronounced in stressful periods.

To investigate whether the extent of transformational leadership differs depending on the situation, the first step of this study is to extend literature by comparing follower perceptions of the same leaders’ transformational behavior in routine versus stressful situations. If followers perceive systematic variations in transformational leadership, this will support the notion that leadership is not invariant, but contingent on the situation. Moreover, by measuring followers’ perceptions how their leaders generally behave under normal versus stressful circumstances, we can estimate the general degree of inconsistency between more or less stressful situations. Our assumption is the following:

*Hypothesis 1a:* The level of transformational leadership is lower in stressful periods than under routine conditions.

Moreover, leaders may not only show less positive behavior under stress, but also lose self-control and show more destructive behavior (Baumeister et al., 1998). In contrast to effects of strain on transformational leadership, effects of leader strain on abusive leadership are more present in the literature. The meta-analysis by Harms et al. (2017) found positive relationships between leaders’ experienced strain and negative behavior targeted at their subordinates. Moreover, abusive leadership has been shown to increase when leaders have to manage challenging work tasks (Mawritz et al., 2014). A study by Li et al. (2016) has found that the crossover effect from leaders’ distress to their followers was mediated by abusive supervision.

In line with this literature, we assume that stressful periods enhance leaders’ impatience and irritability. Lacking positive resources, leaders are expected to display more negative emotional and behavioral reactions and exert less self-control (e.g., Baumeister et al., 1998; Courtright et al., 2016). Consequently, they are expected to show more abusive leadership in stressful than in routine periods:

*Hypothesis 1b:* The level of abusive leadership is higher in stressful periods than under routine conditions.

## Leadership Inconsistency and Follower Health

While there is already some evidence for situational influences on leadership (Halverson et al., 2004; Geier, 2016), research on leadership inconsistency has also come to the fore (Breevaart and Zacher, 2019; Diebig and Bormann, 2020). According to Mullen et al. (2011), leadership inconsistency can be considered as variations of behavior within a leader; the same leader may show different or even contradictory behavior.

One of the first studies investigating leadership inconsistency in terms of contradictory behavior was provided by Duffy et al. (2002). In their cross-sectional study, the authors initially revealed that inconsistent behavior increases employees’ counterproductive behaviors and somatic complaints. The authors argue that inconsistency may evoke feelings of insecurity and a lack of control. In line with this reasoning, de Cremer (2003) showed that inconsistent decision making of leaders is perceived as procedurally unfair and increases employees’ uncertainty, while Mullen et al. (2011) revealed that inconsistency in terms of displaying both transformational and passive leadership predicts followers’ safety participation. Extending research on inconsistent leadership, Breevaart and Zacher (2019) found that inconsistencies in transformational and laissez-faire leadership reduce followers’ trust in the leader. Inconsistent leadership in terms of daily fluctuations in leadership was examined in a recent diary study by Diebig and Bormann (2020). They report a positive relationship between daily laissez-faire leadership variability and daily stress of employees. The authors argue that inconsistent leadership increases followers’ insecurity regarding leader support.

Thus, different forms of leadership inconsistency may influence employees’ health. However, existing studies on inconsistencies either focus on displaying contradictory behavior (Duffy et al., 2002; de Cremer, 2003; Mullen et al., 2011), or refer to short-term daily fluctuations (Breevaart and Zacher, 2019; Diebig and Bormann, 2020). Up to our knowledge, none of the existing studies investigated consequences of global perceptions of inconsistency across situations. Considering the literature, it is likely that not only absolute levels of leadership impact followers, but that also the *extent* of inconsistency (i.e., discrepancies between routine and stressful situations) in leadership *across situations* has a negative effect.

In line with the existing literature, we argue that stronger inconsistencies in transformational and abusive leadership may create ambiguities and uncertainty in followers (de Cremer, 2003; Breevaart and Zacher, 2019; Diebig and Bormann, 2020), which ultimately result in an increase of their strain. Employee strain is an indicator for employee health and comprises their emotional (i.e., irritability) and cognitive (i.e., rumination) irritation (Mohr et al., 2006). We expect that higher leadership discrepancies in routine versus stressful conditions increase followers’ insecurity regarding their leaders’ reliability, support and future actions (Diebig and Bormann, 2020). It might be bearable for employees to endure daily fluctuations in leadership. For example, some leaders display only minor fluctuations with a small range of variation in behavior, to which employees can easily adapt. However, in line with transactional stress theory (Lazarus and Folkman, 1984), we expect that it costs additional psychological capital for employees to adapt to larger-scale differences in leadership. When a leader regularly falters in behavior more strongly, followers must adjust to “different leaders.” When leaders strongly reduce their transformational leadership in stressful periods, followers lack leader support in terms of motivation or individual consideration. Followers then abruptly need to invest more own resources and capacities to compensate for the loss. In contrast, when abusive leadership increases in stressful periods, followers suddenly need to cope with an increasing amount of hostile and aggressive behavior. Employees then immediately need to invest resources (e.g., coping strategies) to protect their health. While inconsistent leaders are likely perceived as unpredictable and unreliable, coping with uncertainty and adapting one’s own behavior to pronounced differences between routine and stressful periods may require higher efforts for followers so that strain will likely increase. Consequently, we expect that higher discrepancies in leadership perceived by followers are related to higher follower strain.

We define the degree of leadership inconsistency as the difference between perceived leadership under routine conditions and under stress for transformational and abusive leadership, respectively. Assuming that followers’ perceptions of transformational leadership are lower and perceptions of abusive leadership are higher in stressful periods, negative difference scores indicate stronger deteriorations for transformational leadership, whereas positive difference scores indicate stronger increases for abusive leadership. We hypothesize the following:

*Hypothesis 2a:* Inconsistencies in transformational leadership are negatively related to follower strain above and beyond the level of transformational leadership.
*Hypothesis 2b:* Inconsistencies in abusive leadership are positively related to follower strain above and beyond the level of abusive leadership.

## Leader Strain and Leadership Inconsistency

We expect that leadership inconsistency is not only related to follower, but also to leader strain. The literature already reports a relationship between leader strain and leadership behavior (Burton et al., 2012; Kaluza et al., 2019). For example, Harms et al. (2017) meta-analyzed the relationship between leader strain and negative as well as positive leadership. While the authors found some support for their assumption that leader strain drains their cognitive and emotional resources so that leaders are not able to function effectively anymore, they were unable to locate studies that report relationships between leader strain and transformational leadership. They found that leaders’ anger and frustration go along with an increase of abusive supervision. Moreover, a review and meta-analytic examination by Kaluza et al. (2019) demonstrates that positive leader well-being is associated with constructive leadership, such as relational and change oriented behaviors, and that poor leader well-being is positively related to destructive leadership such as passive or active destructive behaviors.

However, existing studies did not explicitly investigate the respective associations of explicit differences in leadership in routine versus stressful periods with leader strain. Based on the literature we consider that perceived leader strain is not only related to general perceptions of leadership, but particularly to perceptions of high-level inconsistencies in leadership across situations. If leaders’ level of strain is relevant for leadership discrepancies, higher levels of strain perceived by employees should result in perceptions of more inconsistencies (i.e., stronger differences between routine and stressful conditions), while lower strain levels should be associated with less inconsistencies (i.e., smaller differences between routine and stressful conditions). This would be in line with ego depletion theory, according to which leaders have only limited amounts of resources to exert self-control (Baumeister et al., 1998). For example, when transformational leaders show lower strain levels, they may have enough capacities and resources to engage in positive leadership on a moderate level, even in a stressful situation. However, when leaders suffer from high strain, they may lack resources and capacities to engage in positive behaviors and transformational leadership deteriorates. In turn, when potentially abusive leaders’ strain level increases, they may lack the necessary self-control to avoid negative behavior, so that abusive leadership increases, whereas lower strain levels may help them to restrain themselves and avoid negative behaviors. Consequently, we propose that the more leaders suffer from strain, the more inconsistency there is in their transformational and abusive leadership, respectively. A decrease of transformational leadership is indicated by negative values – i.e., the greater the difference, the lower the inconsistency score, which results in a negative relationship with leader strain. In contrast, an increase of abusive supervision results in positive values – so that the greater the difference, the higher the inconsistency score, which results in a positive relationship with strain. We hypothesize the following:

*Hypothesis 3a:* The level of perceived leader strain is negatively related to differences between routine and stressful situations in transformational leadership.
*Hypothesis 3b:* The level of perceived leader strain is positively related to differences between routine and stressful conditions in abusive leadership.

Figure 1 summarizes the conceptual model: We expect leader strain to be associated with a larger inconsistency in leadership, as illustrated by the arrows from leader strain to the gaps between transformational and abusive leadership under stress and under routine conditions, respectively. In turn, leadership inconsistency is expected to relate to follower strain (i.e., the arrows from the gaps to follower strain), in addition to the general levels of leadership (the latter is illustrated in the arrow from leadership under routine conditions to follower strain).

## Materials and Methods

### Sample and Procedure

Participants were recruited via convenience sampling through the authors’ personal networks to participate in an online survey. The participation was voluntary and anonymous. The sample consisted of *N* = 304 employees from several branches with a mean age of *M* = 32.05 years (*SD* = 10.06) who were in an employment relationship during the survey period. The majority of the sample (77.3%, *N* = 235) were male. Around 37% (*N* = 112) of the participants reported having an academic degree and about 76% (*N* = 233) had permanent jobs. Employees for example worked in public services, the industry sector, education, finance, trading, gastronomy, IT, media, or transport. To investigate the relationships between perceived leadership, follower strain and perceived leader strain, we separately measured transformational and abusive leadership under routine conditions and under stress, as well as leaders’ and followers’ psychological strain.

### Measures

#### Transformational Leadership

For transformational leadership, we used the German version of the Multifactor Leadership Questionnaire (Felfe, 2006b). For reasons of parsimony, a shortened version with 10 items was used. In order to capture the full concept of transformational leadership, the items represented the four subscales: Inspirational motivation (three items, e.g., “*My direct supervisor talks optimistically about the future*”), idealized influence attributed and behavior (with two items each, e.g., “*My direct supervisor makes me proud to be associated with him/her*”), and individualized consideration (three items, e.g., “*My direct supervisor spends time coaching me*”). First, employees rated their leaders’ general transformational behavior (introduction*: “Please indicate in the left column how often a statement is applicable to your direct supervisor in general”)* which depicts routine conditions. Second, employees rated the same items, but for stressful situations (introduction: *“Please indicate in the right column how often the same statement is applicable to your direct supervisor under stress”).* Items were rated on a 5-point Likert scale from 1 = *almost never* to 5 = *almost always.* Cronbach’s alpha was α = 0.93 for both routine and stressful situations.

#### Abusive Supervision

Abusive leadership was measured using the German version of the Abusive Supervision Scale with 13 items by Loock and Schilling (2010). Direct offensive-humiliating behaviors were measured with six items (e.g., “*It may occur that my direct supervisor humiliates employees in front of others*”), and insincere-unfair behaviors were measured with seven items (e.g., “*It may occur that my direct supervisor lies to his/her employees*”). Employees rated their immediate supervisor’s extent of abusive supervision under routine condition and under stress, respectively. Items were rated on a 5-point Likert scale from 1 = *almost never* to 5 = *almost always.* Cronbach’s alpha was α = 0.96 for both routine and stressful periods.

#### Leader Strain

We used leader strain as an indicator for leader health. Employees rated their leaders’ strain with a self-developed scale with four items, based on the Irritation Scale by Mohr et al. (2005). Items were “*My direct supervisor is often irritated*,” “*My direct supervisor often seems tired and tense*,” “*My direct supervisor is often hectic*,” and “*My direct supervisor often complains about stress*” (α = 0.80). Items were rated on a 5-point Likert Scale from 1 = *not at all true* to 5 = *completely true*.

#### Follower Strain

Cognitive irritation was used as an indicator for followers’ psychological health. The Irritation Scale by Mohr et al. (2005) with seven items was used including items such as “*I often feel tired and exhausted*” (α = 0.87). Items were rated on a 5-point Likert scale from 1 = *not at all true* to 5 = *completely true*.

Table 1 shows means, standard deviations and correlations for all study variables.

**TABLE 1:** Relationships between transformational leadership, abusive leadership, leader and follower strain.

	*M*	SD	1	2	3	4	5	6	7	8
1. Transformational leadership routine condition	3.43	0.96	(0.93)							
2. Transformational leadership stress condition	3.12	0.98	0.90**	(0.93)						
3. Differences in transformational leadership^a^	−0.31	0.43	−0.17**	0.27**	−					
4. Abusive supervision routine condition	1.90	0.90	−0.70**	−0.65***	0.07	(0.96)				
5. Abusive supervision stress condition	1.95	0.93	−0.68**	−0.67**	−0.03	0.94**	(0.96)			
6. Differences in abusive supervision^a^	0.05	0.31	−0.00	−0.13*	−0.29**	−0.08	0.26**	−		
7. Follower strain	2.34	0.83	−0.24**	−0.24**	−0.02	0.24**	0.27**	0.13*	(0.87)	
8. Leader strain	2.41	0.90	−0.37**	−0.43**	−0.15*	0.46**	0.49**	0.13*	0.28**	(0.80)

*N = 304.*

*^a^Differences were calculated by subtracting scores under routine conditions from scores under stress conditions.*

**p < 0.01; *p < 0.05.

*Cronbach’s alpha in parentheses across the diagonals.*

## Results

Data was analyzed using SPSS 25. Whereas Hypothesis 1 was tested via *t*-tests, Hypotheses 2a and 2b were tested using hierarchical regression analyses. Hypotheses 3a and 3b were tested by calculating correlation analyses.

Hypothesis 1 postulated that the level of transformational leadership is lower, whereas the level of abusive leadership is higher in stressful periods. *T*-tests for dependent samples were computed to compare the means of the respective leadership constructs under routine conditions vs. stress conditions. Results showed significant differences between routine and stressful conditions. As expected, leaders showed less transformational leadership under stress (*M* = 3.12, *SD* = 0.98) than under routine conditions [*M* = 3.43, *SD* = 0.96; *t*(303) = −12.56; *p* < 0.001]. Moreover, leaders displayed more abusive supervision under stress (*M* = 1.95, *SD* = 0.93) than under routine conditions [*M* = 1.90, *SD* = 0.90; *t*(303) = −2.96; *p* < 0.001]. These findings support Hypotheses 1a and 1b.

Hypothesis 2 postulated that differences in leadership between routine and stress conditions would explain incremental variance in follower strain in addition to general levels of transformational and abusive leadership. To measure leadership inconsistencies between routine and stressful conditions, we subtracted the mean of routine conditions from the mean of stress conditions. This implied negative difference scores for transformational leadership, which was lower under stress, and positive difference scores for abusive leadership, which was higher under stress. Larger differences for both behaviors indicate higher leadership inconsistencies between routine and stressful conditions. The results of the hierarchical regression analysis show that differences in transformational leadership explain additional 0.4% of variance over and above the general level of transformational leadership (Table 2). This increment was not significant (*B* = −0.128, Δ*R*^2^ = 0.004, *p* = 0.243). As shown in Table 3, the analysis for abusive supervision reveals that differences in abusive supervision explain additional 2.4% of variance over and above the general level (*B* = 0.412, Δ*R*^2^ = 0.024, *p* < 0.01). Hypothesis 2b was supported, while Hypothesis 2a was rejected.

**TABLE 2:** Hierarchical regression of follower strain on transformational leadership.

Step	Predictor	Follower strain				
		*B*	*SE B*	β	*R* ^2^	Δ*R*^2^
1	Transformational leadership	−0.207**	0.049	−0.238**	0.054**	0.057**
2	Transformational leadership	−0.217**	0.049	−0.250**	0.055	0.004
	Differences in transformational leadership^a^	−0.128	0.109	−0.066		

*N = 304.*

*^a^Differences were calculated by subtracting scores under routine conditions from scores under stress conditions.*

**p < 0.001.

**TABLE 3:** Hierarchical regression of follower strain on abusive supervision.

Step	Predictor	Follower strain				
		*B*	*SE B*	β	*R* ^2^	Δ*R*^2^
1	Abusive supervision	0.216**	0.052	0.235**	0.052**	0.055**
2	Abusive supervision	0.228**	0.051	0.247**	0.073*	0.024*
	Differences in abusive supervision^a^	0.412**	0.148	0.154**		

*N = 304.*

*^a^Differences were calculated by subtracting scores under routine conditions from scores under stress conditions.*

**p < 0.001, *p < 0.01.

Hypothesis 3 postulated that the level of perceived leader strain is negatively related to differences between stressful and routine conditions (i.e., inconsistencies) in transformational leadership (H3a), and positively related to differences between stressful and routine conditions (i.e., inconsistencies) in abusive leadership (H3b). Table 1 shows the correlations between leaders’ strain and differences (i.e., inconsistencies) in leadership between routine and stressful conditions. We found a negative relationship between leaders’ strain and inconsistencies in transformational leadership (*r* = −0.15, *p* < 0.05), as well as a positive relationship between leaders’ strain and inconsistencies in abusive supervision (*r* = 0.12 *p* < 0.05). Hypothesis 3a and 3b were supported.

## Discussion

The aim of this study was to examine the relationships between inconsistent leadership, follower and leader strain as perceived by followers. Drawing upon previous research (Harms et al., 2017; Kaluza et al., 2019), we expected that leadership would be less favorable under stress such that leaders show less transformational and more abusive leadership. Moreover, we expected that leadership inconsistencies between routine and stressful conditions would be related to both followers’ and leaders’ strain. Results showed systematic differences in perceived leadership, such that followers experience poorer leadership in stressful versus routine times. These inconsistencies were associated with both leader and follower strain, and with regard to abusive leadership also above and beyond the general levels of leadership. Our findings suggest that the quality of leadership is lower when leaders are under stress, and that these inconsistencies may pose an additional stressor for followers which may negatively affect their health.

First, we examined systematic differences in the same leaders’ behavior as perceived by followers. Confirming our hypotheses, the same leaders were perceived as less transformational and more abusive under stressful compared to routine conditions. In stressful periods leaders have to deal with a lack of time as well as a reduction of functional capacity (Hannah et al., 2009; Harms et al., 2017), which may reduce their capability to engage in transformational behavior (Geier, 2016). Moreover, our study uncovered higher levels of abusive leadership under stressful conditions. The lack of resources in stressful periods may lead to a loss of self-control (Baumeister et al., 1998), increasing negative emotional and behavioral reactions toward followers. These findings underline difficulties to maintain consistent leadership in stressful periods. Although the reported differences are rather small, it should be considered that these are only averaged values, and that for some leaders the fluctuation range is large, while for others it is rather small, so that employees are affected by inconsistencies to different degrees. Our study strengthens the evidence for situational influences which may explain leadership inconsistency and extends previous literature by uncovering systematic differences in leadership within leaders across stressful versus routine times.

Second, based on literature on leadership inconsistency (de Cremer, 2003; Mullen et al., 2011), we expected that the perception of the amount of inconsistency (i.e., leadership differences between routine versus stressful conditions), is aversive by itself and contributes to follower strain above and beyond general levels of leadership. In line with our assumption, perceived inconsistency in abusive leadership explained incremental variance in follower strain. Adapting to leadership inconsistency in terms of abusive leadership seems to require additional psychological effort for employees, as they are unsure what behavior to expect at what time (de Cremer, 2003; Diebig and Bormann, 2020). This is in line with previous studies on inconsistent leadership which have shown that inconsistency increases followers’ uncertainty and reduces perceptions of procedural fairness and trust (de Cremer, 2003; Breevaart and Zacher, 2019).

However, in contrast to our assumption, differences in transformational leadership did not explain follower strain. This is puzzling because, on a purely descriptive level, the differences across situations were larger for transformational than for abusive leadership. A possible explanation could be that decreases in positive leadership are not as relevant for follower strain, because employees may be more understanding as they attribute this change to their leaders’ strain. In contrast, leaders who become more abusive under stress may be perceived as passing their strain on to their followers, who likely feel unfairly treated and react themselves with more strain. Alternatively, a decrease of positive behaviors may be not as relevant for negative strain reactions as increased negative leadership, which reflects an additional stressor.

Third, in order to provide additional evidence that leader strain is relevant for leadership discrepancies, we tested whether perceived leader strain is related to perceptions of leadership inconsistency. In line with ego depletion theory (Baumeister et al., 1998), our results show that the more leaders suffer from strain, the more pronounced are inconsistencies between leadership across situations. Higher levels of leader strain were associated with larger differences in transformational and abusive leadership. These findings support the notion that leadership, and especially leadership inconsistency, is contingent on the situation. The phenomenon that the same leader displays fluctuating, sometimes even contradictory behavior has already been addressed as inconsistent leadership in the literature. Yet, this emerging field has so far neglected the respective associations of explicit differences in leadership across situations (Mullen et al., 2011; Breevaart and Zacher, 2019). By establishing a direct link between leader strain and inconsistent behavior within persons, our study provides a first step to understand how leadership inconsistency may come about. Results suggest that leaders’ strain levels are relevant for leadership discrepancies and that higher strain levels are associated with higher discrepancies.

### Theoretical Implications

In line with previous research, our findings support the notion that leadership is contingent on the situation, as perceived transformational leadership was higher and abusive leadership was lower in stressful periods (Geier, 2016; Klebe et al., 2021). Especially inconsistencies in abusive leadership, even on a small scale, seem to affect followers rather than inconsistencies in positive behavior. Our findings have important implications for the emerging research field of leadership inconsistency: First, the fact that followers perceive systematic differences in leadership depending on the presence of stress shows that behavioral styles may not be as stable as research often implies (Montano et al., 2017).

Second, and most importantly, not just the leadership style itself, but also perceptions of consistency seem to be relevant for follower strain, supporting previous findings (Mullen et al., 2011; Breevaart and Zacher, 2019). Still, further theory development is needed to conceptualize consistency and inconsistency in leadership. In our study, leaders differed in the degree of perceived consistency, reflected in the extent of differences between normal and stressful conditions. Yet one may also raise the question whether we still capture *inconsistency* when followers observe systematic differences, as these differences reflect inconsistency across situations, but *consistency* within persons. Thus, our understanding of leadership inconsistency may also reflect a stable inconsistent behavior within persons. In contrast, behavioral changes from leaders that seem completely arbitrary and follow no discernable pattern may reflect a different quality of inconsistent leadership and elicit different reactions.

Furthermore, our findings underline leader strain as a risk factor for leadership quality and consistency, as well as for follower health. Keeping leaders in a good state of health may not only lead to a stabilization of leadership, but also prevent followers from experiencing negative leadership and in turn psychological strain. In order to better understand how and why leader strain may lead to deteriorating leadership, further theory development should take a differentiated view on specific stressors, such as time pressure or the general workload of leaders to identify potential risk situations and to enable leaders and organizations to counteract at an early stage.

### Strengths, Limitations, and Recommendations for Future Research

The present study has some limitations that should be considered when interpreting the results. First, the cross-sectional design is a limitation regarding causality. Although we can reasonably assume that leader strain influences behavior, we cannot rule out that strained followers are more likely to perceive poorer leadership, or that leaders’ behavior reflects a reaction to conflicts with strained followers. However, though this line of reasoning applies to the general stressor-strain relationship, we see no theoretical or empirical reason why high strain might prompt followers to perceive stronger *differences* in leader behavior across situations.

Additionally, we measured followers’ perceptions of differences by comparing leadership ratings for stressful and routine situations. We cannot rule out that these perceptions were influenced by follower characteristics, attributions or recall biases. On the other hand, these simultaneous overall ratings have the advantage that followers can draw on all past experiences of stressful situations with their leaders and provide a representative picture. Drawing upon measurements at specific points of time may increase the risk of observing untypical situations and behavior. Nevertheless, future studies should employ week-level designs to investigate changes in leader behavior, stress and strain over time and test the direction of effects.

Second, the present study may be affected by common method bias (Podsakoff et al., 2003), since all variables were rated by employees. Future studies should collect data from different perspectives, so that leaders and followers each rate their own strain, and complement our design with objective strain indicators, such as heart rate variabilities or absenteeism. Nevertheless, it is important to acknowledge the relevance of employees’ perceptions of leadership (Perko et al., 2016). Follower perceptions are particularly relevant in the context of strain. It is likely that subjective perceptions of inconsistency and the associated rumination contribute to follower health, which may be more relevant than any “objective” degree of leadership deterioration. This is in line with stress theories emphasizing subjective appraisal processes (Lazarus and Folkman, 1984). This reasoning could be explicitly tested in future studies contrasting effects of follower perceptions with self-reports from leaders or observer ratings of leadership.

Related to issues of causality and measurement, it should be noted that some reported effects are rather small, especially the difference in abusive leadership. However, behavior and well-being at work are complex and determined by a multitude of factors, so that observed effects of any single variable cannot be large. Even small effects can have meaningful consequences when they represent rather large differences in relative risks (Mohr and Semmer, 2002). Moreover, in addition to inflated correlations due to common method bias, there are several mechanisms in the stressor-strain relationship that may lead to an under-estimation of effects, such as moderator variables or selection effects (Zapf et al., 1996). In the present study, it is possible that followers underestimated the differences in abusive behavior, because they were asked to think about stressful versus normal work situations in general and may have had difficulties to recall such situations and behavior. Especially when abusive behavior is less frequent for a given leader, these difference ratings may be positively biased. As results have demonstrated, even a small increase in abusive supervision was related to follower strain, so that even a small increase of negative leadership behavior may be more crucial than any decrease of positive behavior.

### Practical Implications

Regarding practical implications, findings suggest that organizations should be aware of the links between leadership and strain and consequently consider leader health in personnel development efforts. Organizations should make leaders aware of their responsibilities for their own as well as their followers’ health, including potential crossover of their own strain (Franke et al., 2014; Krick et al., 2021). Hence, organizations should ideally integrate leadership trainings with occupational health promotion (Kelloway and Barling, 2010), fostering leaders’ health awareness and well-being. Our findings indicate that problems related to strain crossover to employees, and poor leadership cannot simply be resolved by selecting stress-resistant persons for leadership positions. By investing in leaders’ health and their resources to cope with stress appropriately, organizations can help to prevent deteriorating leadership and inconsistencies, which would also account for follower health.

## Conclusion

The present study is the first to connect systematic differences in leadership across situations to both follower and leader strain. We identified deteriorations in leadership in stressful periods, and we uncovered relationships between leadership inconsistency and both followers’ and leaders’ strain. Most importantly, differences in abusive leadership were positively related to follower strain in addition to the general level of abusive supervision, underlining inconsistency in leadership as a stressor in its own right. Moreover, followers experience less positive leadership and more abusive leadership when leaders are strained. Results suggest that in order to protect follower health, organizations should aim at supporting leaders in stressful periods and thus strengthen leadership consistency.

## Data Availability Statement

The raw data supporting the conclusions of this article will be made available by the authors, without undue reservation.

## Ethics Statement

Ethical review and approval was not required for the study on human participants in accordance with the Local Legislation and Institutional Requirements. The patients/participants provided their written informed consent to participate in this study.

## Author Contributions

LK and JF developed the research question and study design. LK performed the statistical analyses and provided a first version of the manuscript, closely supported by KK. JF provided the data and supervised the writing process. All authors discussed the results and contributed to the final manuscript.

## Conflict of Interest

The authors declare that the research was conducted in the absence of any commercial or financial relationships that could be construed as a potential conflict of interest.

## Publisher’s Note

All claims expressed in this article are solely those of the authors and do not necessarily represent those of their affiliated organizations, or those of the publisher, the editors and the reviewers. Any product that may be evaluated in this article, or claim that may be made by its manufacturer, is not guaranteed or endorsed by the publisher.
